# Use of mobile phone in operating room

**DOI:** 10.4103/0971-6203.51938

**Published:** 2009

**Authors:** Sanjay Saraf

**Affiliations:** Department of Plastic Surgery, NMC Specialty Hospital, Dubai, U.A.E. E-mail: drsaraf@hotmail.com

Mobile phones and Personal Digital Assistants (PDAs) have become an integral part of the physician's life. They are commonly used for personal and professional scheduling, accessing medical information, drug information and e mails.

Contrary to the belief, there is a common practice to use mobiles and PDAs by surgeons in Operating Room (OR) both for personal and professional use. Surgeons commonly use mobile cameras to take photographs intra-operatively in case of non-availability of cameras. Many of the surgeons use mobiles and PDAs to play live or recorded music during surgeries. A study found 66% of surgeons using their phones in the hospitals, including in operating theatres and intensive care units.[1]

Regarding mobile usage in OT, the question of safety is complex and in practice, often confusing. Lot of concerns has been raised regarding the interference of the vital apparatus in OT and ICU. Many Anecdotal reports exist of interference with medical electrical equipments.[2,3] However; it is difficult to compare various study findings due to number of devices tested, non-uniform study designs and heterogeneous technological information.

The issues concerning mobile phones is the electromagnetic radiation (EMR) they produce, the safe distance they should be from medical equipment and the ability of medical equipment to resist EMR. Another issue is - noise from mobile phones ringing during consultation or in the ward disturbing staff and irritating patients.

The clinically relevant EMR is suggested to be the interference sufficiently altering the operation of medical equipment endangering a patient. This includes switching off, faulty action and interrupted function of equipment. The studies have found that most of the interference related to disturbance of the signals is on cardiac monitors.[4] Other effects were found on pacemakers, with inappropriate inhibition or atrial over sensing or misinterpretation of the mobile phone signal as atrial activity with synchronous fast pacing of the ventricle, documented in both permanent and temporary systems.[5,6] The effect on both devices was, however, found to be transitory and completely avoidable by removing the mobile phone away from the monitor or pacemaker. Moreover, the interference with the pacemaker occurred only with the mobile phone at a distance of up to 10 cm from the equipment.[6]Studies by Medical Devices Agency (UK) also revealed clinically relevant electromagnetic interference with the usage of mobile phones in four per cent of medical electrical equipment, though testing was not standardized between studies and equipment tested were not identical. The studies recommend some type of restriction of mobile phone use in hospitals, with use greater than one meter from equipment. The newer generation mobile phones might be used much closer to other equipment than their predecessors. However, the safest option is the “one meter rule” proposed by Irnich and Tobisch which suggested restriction of mobile phone use to greater than 1 meter from equipment.[7]

Most world standards relate to the United States Food and Drug Administration (FDA), which issued a voluntary standard in 1979 specifying that medical equipment should be immune from interference in fields of up to 7 V/m within the frequency range of 450–1000 MHz.^2^ Clearly, mobile phones may breach these limits if operated within one meter, but this does not automatically translate to EMI.[2] In Europe, Asia and Middle East, phones operate predominantly in the 1800 MHz band. Mobile phones operating at 1800 MHz appear to cause clinically non significant EMI and need to be closer to medical equipment to affect it. Recent introduction of Bluetooth technology offers further less interference.

A study by Edelstyn and Oldershaw investigating the effects of acute mobile phone exposure on attention system found that performance was facilitated following mobile phone exposure with no deficits evident. Another study by Lee *et al*, on the effect of mobile phones on human attention suggested that exposure to the electromagnetic field emitted by mobile phones may have a mild facilitating effect on attention functions and also on cognitive processing.

With technological advancement, newer equipment is becoming less sensitive to interference as manufacturers are adopting increasingly stringent standards for screening.[8]

Immediate access to medical information at the point of care can reduce costs, improve accuracy in diagnosis and treatment, reduce errors, and optimize workflow.[9] Short message service (SMS) is also a valuable asset for surgeons to discuss management of interesting and difficult cases with their peer. An Australian study by Lam *et al*. in 2004 utilized mobile phone photo-messaging in 27 cases of hand trauma for communication between the registrar and the consultant in the emergency department and recommended the use of mobile phone photo messaging into the clinical practice.

The Medicines and Healthcare Product Regulatory Agency of United Kingdom recommends that ‘a balanced approach is necessary to ensure that the benefits of mobile wireless technology can be made to all organizations’.[1]

The uniform guidelines need to be evolved to avoid potential litigation. Further research on clinically significant electromagnetic interference in medical equipment from mobile phones needs to be done. The medical equipment need to be manufactured to resist electromagnetic interference from mobile phones, with standards that take into account the new phone technology.

Although safety of mobile phones in operating rooms is a complex issue, according to recent literature, the risk of electromagnetic interference from mobile phones appears to be minimal and controllable. There is no clinically relevant electromagnetic interference as long as they are more than a meter away from sensitive equipments. They are not as hazardous as believed and we will have to adopt a more sensible, evidence-based balanced policy towards mobile phone usage in our clinical practice.
